# Longterm reduction rate in catheter related bloodstream infections after implementation of a new strategy

**DOI:** 10.1186/2047-2994-4-S1-O25

**Published:** 2015-06-16

**Authors:** M Logar, M Prosen, T Lejko Zupanc

**Affiliations:** 1 Department of Infectious Diseases, University Medical Centre Ljubljana, Ljubljana, Slovenia; 2 Hospital Hygiene, University Medical Centre Ljubljana, Ljubljana, Slovenia

## Introduction

Catheter-related bloodstream infection (CRBSI) accounts for 10% to 20% of hospital-acquired infections and is associated with both increased ICU stay and mortality. CRBSI represent a considerable toll on patients and hospital resources. A lot of effort is put into the prevention of CRBSI.[1].

## Objectives

The aim of the present report is to evaluate the long-term results of the implementation of the strategies for prevention of catheter related infections.

## Methods

Standardized questionnaire for the evaluation of CRBSI was implemented to the ICU in University medical center Ljubljana in 2010. After the observation period we evaluated the rate of CRBSI in different ICUs. At the end of the observation period we started with the implementation of the strategies for prevention of catheter related infections. Part of this strategies was also participation in PROHIBIT study. Central line insertion and care bundles were implemented together with the education about the hand hygiene according to the WHO campaign “5 moments of hand hygiene”. After the end of PROHIBIT study we continue to perform regular surveillance of CRBSI in ICUs on daily basis together with ICU staff. Analysis of the date is done twice a year and date is returned to the ICUs. Additional education or interventions are performed as needed according to the results.

## Results

In 2010 the mean incidence of CRBSI in our institution was 5,6 CRBSI/1000 catheter days (CD). After the implementation of strategies for prevention of catheter related infections the incidence dropped dramatically in all ICU, but there were still some differences between ICUs. The lowest combine incidence for all ICUs was 0.67 CRBSI/1000 CD. After the end of PROHIBIT study the incidence has risen slightly but after the implementation of regular surveillance the incidence dropped again and was 0.63 CRBSI/1000 CD in 2014.

## Conclusion

Our experience in prevention of CRBSI shows it is possible to maintain low rate of CRBSI also after the study period without big expenses but with regular surveillance with feedback information and action according to the results.

## Disclosure of interest

None declared.
